# Knowledge of tuberculosis among female sex workers in Rajshahi city, Bangladesh: a cross sectional study

**DOI:** 10.1186/s12879-019-4531-0

**Published:** 2019-10-11

**Authors:** Md. Masud Rana, Md. Rafiqul Islam, Sheikh MoinUddin, Md. Abdul Wadood, Md. Golam Hossain

**Affiliations:** 1 Department of Population Science and Human Resource Development, University of Rajshahi, Rajshahi, 6205 Bangladesh; 2 Faridpur TB and Leprosy Control Project, Damien Foundation, Faridpur, Bangladesh; 3 Rajshahi University Medical Center, University of Rajshahi, Rajshahi, 6205 Bangladesh; 4 Department of Statistics, University of Rajshahi, Rajshahi, 6205 Bangladesh

**Keywords:** Tuberculosis, Female sex-workers, Rajshahi, Bangladesh, Chi-square test

## Abstract

**Background:**

Tuberculosis (TB) is a major public health problem in developing countries like Bangladesh. Female sex workers (FSWs) and their clients are active sources for spreading TB. The purpose of this study was to assess the knowledge of TB among FSWs in Rajshahi city, Bangladesh.

**Methods:**

It was a cross-sectional study with a sample size of 225 FSWs. The knowledge on TB was measured by six different questions. Chi-square test and multinomial logistic regression model were used in this study to find the associated factors of lack of general knowledge on TB among FSWs.

**Results:**

Out of 225 FSWs, 43.1, 34.7 and 22.2% came from urban, rural and slum areas respectively. More than 41% FSWs perceived that TB is a non-communicable disease. A large number of FSWs (76.4%) did not know the spread of TB. It was found that more than 90% FSWs did not have knowledge on latent TB. The χ_2_-test demonstrated that FSWs’ education, monthly family income, age, currently marital status and sex trading place were significantly associated with their knowledge on TB. A remarkable number of FWSs (42.2%) had poor knowledge on TB. It was found that comparatively higher educated FWSs were more likely to have good or fair knowledge on TB than lower educated ones (*p* < 0.01).

**Conclusions:**

This study revealed that near to half of FSWs in Rajshahi city, Bangladesh had poor knowledge on TB. Public health authorities should pay due attention and adopt policy for increasing the knowledge on TB among FSWs to reduce the incidence of TB in Bangladesh. Subsequently, advocacy, communication for social mobilization program is very urgent.

## Background

Tuberculosis (TB) is still a burning issue in developing countries like Bangladesh, where favorable environment is available for the spread of TB. Female sex workers (FSWs) are most important vulnerable groups for getting and spreading TB, because they trade sex with their clients without any information of clients’ TB status in Bangladesh [1]. It was estimated in 2009 that the number of FSWs was 63 to 74 thousand in Bangladesh [2]. Usually, FSWs in Bangladesh trade sex in street, hotel, residence and brothel. Hotel and residence based FSWs entertained an average of 61 clients per week in Bangladesh [2]. There are few brothels in Bangladesh, and Rajshahi is one of the cities where there is no brothel. As sex trade is illegal outside of brothel in Bangladesh and it is strongly considered as antisocial activities, it is a great challenge for FSWs in this country to run their trade. They are frequently bound to change their identities including their names, addresses, cell phone numbers etc. It was reported that the number of hotel and residence based FSWs in Rajshahi city was 40.5 and 39.0% respectively, and a remarkable number (20.5%) of FSWs trade sex in street [2]. Most of the sex trading places are unhygienic and favorable environment for TB. Besides, the street is comparatively a more vulnerable place for TB bacteria, and most of their clients are transport workers and rickshaw pullers [3]. Rajshahi is one of the biggest cities in Bangladesh, situated at the western border of Bangladesh, separated from India by a branch of Ganges River (Padma branch). Every day many people are travelling India legally or illegally for various purposes, and India is the first ranking country among 30 highly burden countries for TB in the world [4, 5], also the highest prevalence of HIV/AIDS in India among SARC countries [6]. It is important to survey the knowledge on TB among FSWs who are trading sex in this border city. However, some studies have been conducted on TB with other populations such as, community people and key public [7, 8], TB patients [9, 10] and school students [11, 12]. Some other studies were done with medical students, health care workers and people of other occupations [13–15]. Study on TB with medical, nursing and midwifery students were also conducted in other population [16, 17]. According to world health organization 2016, Bangladesh is one of the most highly burden countries out of 30 highly TB burden countries and its rank is 7 with annual occurrence of 362,000 new TB cases [18]. About 73,000 people die annually due to TB in this country. Another important challenge is Multi Drug Resistance TB (MDR TB) - with an estimated 9,700 MDR cases per year [18]. This type of study would be interested for improving policy to control TB in Bangladesh. Survey on knowledge about TB among non-medical university students, industrial labors in Rajshahi have been done [19, 20]. These studies neither identified FSWs nor focused on association of TB with them, and did not examine risks of the easy spread of TB by the FSWs. To the best of our knowledge there is no other report or study about the knowledge on TB among FSWs in Bangladesh, however one study has been done with FSWs in Rajshahi city to survey their knowledge on HIV/AIDS [2]. As association between the knowledge on TB among FSWs is very important because socio-economic and demographic factors of them and as well as their clients, and eventually the community as a whole, are largely affected by TB. Though our study was conducted in Rajshahi city, it would explore some aspects of risks and help finding out measures to save the FSWs, their clients and the society as a whole from TB in Bangladesh.

The aim of the present study was to investigate the knowledge on TB and its associated factors of FSWs in Rajshahi city, Bangladesh.

## Methods

### Study design and population

**Fig. 1:**
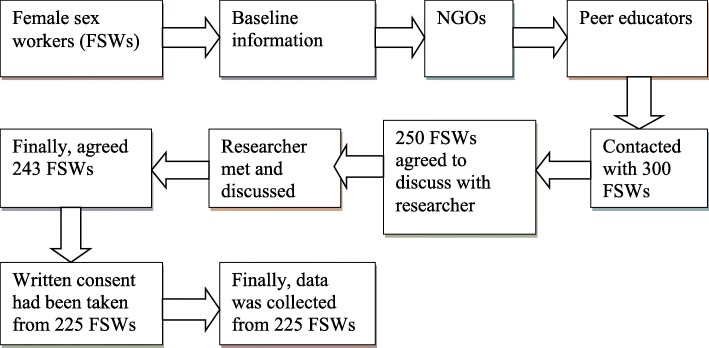
Sample selection procedure

It was a cross-sectional study. The sample consisted of 225 FSWs who traded sex at different places such as streets, residences and hotels in Rajshahi city, Bangladesh. This study was based on the complete good clinical procedures. FSWs’ personal cell phone numbers were collected from the NGOs working for welfare of FSWs and their clients. Primarily we contacted the key personnel of the NGOs. They introduced us with their peer educators who themselves were FSWs and paid workers of the respective NGOs. These peer educators managed appointments and meetings for us with FSWs. With the help of peer educators we collected 300 FSWs’ personal mobile phone numbers. We contacted 300 FSWs by using mobile phone. Out of them 243 FSWs were willingly agreed for interviews. Finally, 225 FSWs provided their written consent (Fig. 1). Selected FSWs were interviewed at their suggested venue. We followed the procedure which was used in previous study [2].

### Data collection

Study data was collected in the period from July’2015 to December’2016. All of the respondents were street, hotel and residence based FSWs in Rajshahi city. The study design was to document the socio economic, demographic and sex trade practices related to TB knowledge of FSWs and particular attention was given to identify issues related to knowledge assessment of FSWs on TB. The following data were collected for the study:

(i) general and specific information of TB, (ii) socio-economic and demographic characteristics of FSWs, (iii) knowledge on TB. The data were collected by using a semi-structured questionnaire. The original questionnaire was prepared in English but it was translated to Bengali the mother language of Bangladeshi for easy understanding of the subjects. The original and translated questionnaires were reviewed by ten professional experts and volunteers, and a pilot study was conducted to validate the questionnaire. We also modified the questionnaire based on the results of the piloting to make it easier to comprehend and answer. To ensure strictly confidentiality the names of the respondents were not recorded to avoid link from data.

### Outcome variables

The dependent variable in this study was knowledge on TB, which was measured by six dissimilar questions, namely: i) Is TB a communicable disease? ii) Have you ever heard about latent TB? iii) Do you know the main sign and symptom of TB? iv) Do you know how TB spread? v) Do you think is TB curable? vi) Do you know which part is mostly affected by TB? The overall level of knowledge on TB was measured on the basis of the number of correct answers of these six questions; (i) poor knowledge (0–2 correct answers), (ii) fair knowledge (3–4 correct answers) and (iii) good knowledge (5–6 correct answers).

### Independent variables

In this study, theoretically pertinent socio-economic and demographic factors were included as independent variables. We classified age into two groups: a younger group (≤25 years), and an older age group (≥26 years). Education was classified based on the formal education system in Bangladesh: Illiterate (0 years), primary education (1–5 years), secondary and higher education (6 years or more). Marital status was categorized as unmarried and ever married. Place of residence was categorized as rural, urban and slum. Type of family was categorized as single and joint family. Types of clients were categorized as transport workers, small traders and service holder. Sex trade place was categorized as residence, hotel and street. Respondent’s monthly income was categorized as < 8000 BDT and ≥ 8001 BDT.

### Statistical analyses

Data were cross-checked for consistency before final data entry using Microsoft Excel. Descriptive analyses were conducted to determine the socio- economic and demographic factors related with knowledge on TB of the respondents. Chi-square test and Fisher’s exact test (if the cell frequency less than 5) were used to find the association between two factors. Since the level of knowledge on TB among FSWs was of three categories; (i) poor, (ii) fair and (iii) good, it was an outcome variable, multinomial logistic model was selected to determine the effect of socio-economic and demographic factors on overall knowledge of TB. Analyses were performed using statistical package for social sciences (SPSS version 22 IBM). Significance for all analyses was set at *p* < 0.05.

## Results

In this study, we assessed the knowledge on TB among FSWs in Rajshahi city, Bangladesh.

Table 1 shows the socio-economic and demographic profile of FSWs. Out of the total sample population 225 FSWs, more than 54% were above 26 years of age. By education, 14.2% were illiterate, 48.9% were got primary education and the remaining 36.9% had secondary or higher level of education. Out of them, 92.4% were ever married. 43.1% FSWs lived in urban, 34.7% in rural and 22.2% in slum areas. Most of the FSWs (96.4%) came from single family. Out of FSWs’ clients, 63.1% were small trades, while 13.8% transport workers and 23.1% service holders. FSWs’ places of sex trade were hotel (64.4%), residence (11.6%) and street (24.0%). 69.3% FSWs’ monthly income was below 8000 BDT and 30.7% above 8000 BDT. More than 41% FSWs perceived TB as a non-communicable disease. Regarding the knowledge on TB as communicable disease, secondary and higher education (75.9%) and monthly income of ≥8000 BDT (69.6%) of the respondents had higher percentage and than their counterparts, and χ^2^-test demonstrated that the association knowledge on TB and education (*p* < 0.05) and monthly income (*p* < 0.01) were significant (Table 2). A very few number of FSWs (9.8%) heard the term of latent TB, and the association between the knowledge on latent TB and education and monthly family income were statistically significant (*p* < 0.05) (Table 3). It was found that more than 13% FSWs did not have idea any about the main sign and symptoms of TB. More than 87% FSWs believed that cough for 3 weeks or more was the main sign and symptom of TB. The association between the knowledge on sign and symptom of TB and FSWs marital status was significant (*p* < 0.05) (Table 4). Only 23.6% FSWs knew about the spread of TB. On the knowledge of TB spreading, FSWs with secondary and higher education and sex trade at residence had higher percentage (36.1%) and (34.6%) than their counterparts and the association between the knowledge on mode of TB spreading and respondents’ education (*p* < 0.01) and sex trade place (*p* < 0.05) were statistically significant (Table 5). More than 78% respondents strongly believed that TB was a curable disease, and younger and respondents with monthly income ≥8000 in taka had higher percentages (84.5%) (91.3%) than their counterparts and the association between the knowledge on curability of TB and respondents’ age (*p* < 0.05) and monthly family income (*p* < 0.01) were statistically significant (Table 6). More than 81% FSWs did not know which parts of the body are mostly affected by TB. Regarding the knowledge on which parts of the body are mostly affected by TB, secondary and higher educated FSWs had higher percentage (28.9%) than their counterparts and the association between the knowledge of which parts of the body mostly affected by TB and education was statistically significant (*p* < 0.01) (Table 7).

**Table 1:** Socio- economic and demographic profile of female sex workers

Variables	N (%)
Age in years	
≤ 25	103(45.8)
≥ 26	122 (54.2)
Educational status	
Illiterate	32(14.2)
Primary	110 (48.9)
Secondary and higher	83(36.9)
Marital status	
Ever married	208(92.4)
Unmarried	17(7.6)
Type of residence	
Rural	78(34.7)
Urban	97(43.1)
Slum	50(22.2)
Type of family	
Single	217(96.4)
Joint	8(3.6)
Type of client	
Transport workers	31(13.8)
Small traders	142(63.1)
Service holder	52(23.1)
Sex trade place	
Residence	26(11.6)
Hotel	145(64.4)
Street	54(24.0)
Monthly income in BDT	
< 8000	156(69.3)
≥ 8000	69(30.7)

**Table 2:** Knowledge about type of TB disease and its association with socio economic and demographic factors

Is TB communicable disease?				
Variables	No 94(41.8%)	Yes 131(58.2%)		
	N (%)	N (%)	χ^2^/Fisher exact value	*p*-value
Age in years			1.160	0.282
≤ 25	47(45.6)	56(54.4)		
≥ 26	47(38.5)	75(61.5)		
Educational status				
Illiterate	22(68.8)	10(31.2)	21.604	0.001
Primary	52(47.3)	58(52.7)		
Secondary and higher	20(24.1)	63(75.9)		
Marital status			1.156	0.282
Ever married	89(42.8)	119(57.2)		
Unmarried	05(29.4)	12(7.6)		
Type of residence			1.959	0.376
Rural	28(35.9)	50(64.1)		
Urban	45(46.4)	52(53.6)		
Slum	21(42.0)	29(58.0)		
Type of family			0.960	0.327
Single	92(42.4)	125(57.6)		
Joint	2(25.0)	6(75.0)		
Type of client			2.107	0.349
Transport workers	11(35.5)	20(64.5)		
Small traders	57(40.1)	85(59.9)		
Service holder	26(50.0)	26(50.0)		
Sex trade place			1.207	0.547
Residence	10(38.5)	16(61.5)		
Hotel	58(40.0)	87(60.0)		
Street	26(48.1)	28(51.9)		
Monthly income in BDT			5.264	0.022
< 8000	73(46.8)	83(53.2)		
≥ 8000	21(30.4)	48(69.6)

**Table 3:** Knowledge about latent TB and its association with socio economic and demographic factors

Have you ever heard about latent TB?				
Variables	No 203(90.2%)	Yes 22 (9.8%)		
	N (%)	N (%)	χ^2^/Fisher exact value	*p*-value
Age in years			0.001	0.974
≤ 25	93(93.3)	10(9.7)		
≥ 26	110(90.2)	12(9.8)		
Educational status			5.646	0.040
Illiterate	31(96.9)	1(3.1)		
Primary	102(92.7)	08(7.3)		
Secondary and higher	70(84.3)	13(15.7)		
Marital status			0.082	0.774
Ever married	188(90.4)	20(9.6)		
Unmarried	15(88.2)	2(11.8)		
Type of residence			2.638	0.267
Rural	72(92.3)	06(7.7)		
Urban	84(86.6)	13(13.4)		
Slum	47(94.0)	03(6.0)		
Type of family			0.899	0.343
Single	195(89.9)	22(10.1)		
Joint	08(100.0)	0(0.0)		
Type of client			3.815	0.148
Transport workers	29(93.5)	02(6.5)		
Small traders	124(87.3)	18(12.7)		
Service holder	50(96.2)	02(3.8)		
Sex trade place			3.727	0.155
Residence	21(80.8)	05(19.2)		
Hotel	131(90.3)	14(9.7)		
Street	51(94.4)	03(5.6)		
Monthly income in BDT			3.326	0.042
< 8000	137(87.8)	19(12.2)		
≥ 8000	66(95.7)	03(4.3)

**Table 4:** Knowledge about sign and symptom of TB and its association with socio economic and demographic factors

Do you know main sign and symptom of TB?				
Variables	No 30(13.3%)	Yes 195 (87.7%)		
	N (%)	N (%)	χ^2^/Fisher exact value	*p*-value
Age in years			0.083	0.773
≤ 25	13(12.6)	90(87.4)		
≥ 26	17(13.9)	105(86.1)		
Educational status			2.965	0.227
Illiterate	06(18.8)	26(81.3)		
Primary	17(15.5)	93(84.5)		
Secondary and higher	07(8.4)	76(91.6)		
Marital status			2.829	0.043
Ever married	30(14.4)	178(85.6)		
Unmarried	0(0.0)	17(100.0)		
Type of residence			2.640	0.267
Rural	08(10.3)	70(89.7)		
Urban	12(12.4)	85(87.6)		
Slum	10(20.0)	40(80.0)		
Type of family			0.005	0.944
Single	29(13.4)	188(86.6)		
Joint	01(12.5)	07(87.5)		
Type of client			0.219	0.896
Transport workers	04(12.9)	27(87.1)		
Small traders	20(14.1)	122(85.9)		
Service holder	06(11.5)	46(88.5)		
Sex trade place			0.303	0.859
Residence	04(15.4)	22(84.6)		
Hotel	18(12.4)	127(87.6)		
Street	08(14.8)	46(85.2)		
Monthly income in BDT			1.852	0.174
< 8000	24(15.4)	132(84.6)		
≥ 8000	06(8.7)	63(91.3)

**Table 5:** Knowledge about TB transmission and its association with socio economic and demographic factors

Do you know how to spread TB?				
Variables	No 172(76.4%)	Yes 53 (23.6%)		
	N (%)	N (%)	χ^2^/Fisher exact value	*p*-value
Age in years			1.806	0.179
≤ 25	83(80.6)	20(19.4)		
≥ 26	89(73.0)	33(27.0)		
Educational status			13.845	0.001
Illiterate	30(93.8)	02(6.3)		
Primary	89(80.9)	21(19.1)		
Secondary and higher	53(63.9)	30(36.1)		
Marital status			0.000	0.998
Ever married	159(76.4)	49(23.6)		
Unmarried	13(76.5)	4(23.5)		
Type of residence			3.343	0.188
Rural	64(82.1)	14(17.9)		
Urban	74(76.3)	23(23.7)		
Slum	34(68.0)	16(32.0)		
Type of family			0.010	0.922
Single	166(76.5)	51(23.5)		
Joint	06(75.0)	02(25.0)		
Type of client			1.637	0.441
Transport workers	24(77.4)	07(22.6)		
Small traders	105(73.9)	37(26.1)		
Service holder	43(82.7)	09(17.3)		
Sex trade place			5.441	0.041
Residence	17(65.4)	09(34.6)		
Hotel	108(74.5)	37(25.5)		
Street	47(87.0)	07(13.0)		
Monthly income in BDT			1.229	0.268
< 8000	116(74.4)	40(25.6)		
≥ 8000	56(81.2)	13(18.8)

**Table 6:** Knowledge about the curable of TB and its association with socio economic and demographic factors

Do you think TB is curable?				
Variables	No 48(21.3%)	Yes 177 (78.7%)		
	N (%)	N (%)	χ^2^/Fisher exact value	*p*-value
Age in years			3.807	0.040
≤ 25	16(15.5)	87(84.5)		
≥ 26	32(26.2)	90(73.8)		
Educational status			2.413	0.299
Illiterate	10(31.3)	22(68.8)		
Primary	23(20.9)	87(79.1)		
Secondary and higher	15(18.1)	68(81.9)		
Marital status			0.053	0.818
Ever married	44(21.2)	164(78.8)		
Unmarried	04(23.5)	13(76.5)		
Type of residence			1.551	0.460
Rural	13(16.7)	65(83.3)		
Urban	23(23.7)	74(76.3)		
Slum	12(24.0)	38(76.0)		
Type of family			0.386	0.535
Single	47(21.7)	170(78.3)		
Joint	01(12.5)	07(87.5)		
Type of client			3.797	0.150
Transport workers	10(32.3)	21(67.7)		
Small traders	25(17.6)	117(82.4)		
Service holder	13(25.0)	39(75.0)		
Sex trade place			0.550	0.760
Residence	07(26.9)	19(73.1)		
Hotel	30(20.7)	115(79.3)		
Street	11(20.4)	43(79.6)		
Monthly income in BDT			9.471	0.002
< 8000	42(26.9)	114(73.1)		
≥ 8000	06(8.7)	63(91.3)

**Table 7:** Knowledge about mostly affected part of body by TB and its association with socio economic and demographic factors

Do you know which part of body mostly affected by TB?				
Variables	No 183(81.3%)	Yes (Lung) 42 (18.7%)		
	N (%)	N (%)	χ^2^/Fisher exact value	*p*-value
Age in years			1.679	0.195
≤ 25	80(77.7)	23(22.3)		
≥ 26	103(84.4)	19(15.6)		
Educational status			13.471	0.001
Illiterate	32(100.0)	0(0.0)		
Primary	92(83.6)	18(16.4)		
Secondary and higher	59(71.1)	24(28.9)		
Marital status			0.286	0.593
Ever married	170(81.7)	38(18.3)		
Unmarried	13(76.5)	04(23.5)		
Type of residence			4.348	0.114
Rural	68(87.2)	10(12.8)		
Urban	73(75.3)	24(24.7)		
Slum	42(84.0)	08(16.0)		
Type of family			1.938	0.164
Single	178(82.0)	39(18.0)		
Joint	05(62.5)	03(37.5)		
Type of client			0.355	0.837
Transport workers	26(83.9)	05(16.1)		
Small traders	116(81.7)	26(18.3)		
Service holder	41(78.8)	11(21.2)		
Sex trade place			3.666	0.160
Residence	18(69.2)	08(30.8)		
Hotel	118(81.4)	27(18.6)		
Street	47(87.0)	07(13.0)		
Monthly income in BDT			1.340	0.247
< 8000	130(83.3)	26(16.7)		
≥ 8000	53(76.8)	16(23.2)		

It was observed that 42.2% FSWs had overall poor knowledge on TB while 7.8% had good knowledge. More than 50% FSWs had fair knowledge on TB (Table 8). It was found that the level of poor knowledge decreased with increasing FSWs’ education level. However the level of fair and good knowledge increased with increasing their education level. The association between the level of the knowledge of FSWs and their education level was significant (*p* < 0.01). The other selected factors did not show significant association with the level of the knowledge on TB (Table 8).

**Table 8:** Association between level of knowledge and socio economic and demographic factors among FSWs

Level of knowledge about TB					
Variables	Poor, 95(42.2%)	Fair, 113 (50.2%)	Good, 17(7.6%)		
	N (%)	N (%)	N (%)	χ^2^/Fisher exact value	*p*-value
Age in years					
≤ 25	43(41.7)	55(53.4)	5(4.9)	2.226	0.329
≥ 26	52(42.6)	58(47.5)	12(9.8)		
Educational status					
Illiterate	26(81.2)	5(15.6)	1(3.1)	36.209	0.0001
Primary	49(44.5)	57(51.8)	4(3.6)		
Secondary and higher	20(24.1)	51(61.4)	12(14.5)		
Marital status					
Ever married	89(42.8)	104(50.0)	15(50.0)	0.667	0.716
Unmarried	6(35.3)	9(52.9)	2(11.8)		
Type of residence					
Rural	30(38.5)	44(56.4)	4(5.1)	3.126	0.537
Urban	42(43.3)	45(46.4)	10(10.3)		
Slum	23(46.0)	24(48.0)	3(6.0)		
Type of family					
Single	93(42.9)	108(49.8)	16(7.4)	1.100	0.577
Joint	2(25.0)	5(62.5)	1(12.5)		
Type of client					
Transport workers	14(45.2)	16(51.6)	1(3.2)	5.012	0.286
Small traders	54(38.0)	74(52.1)	14(9.9)		
Service holder	27(51.9)	23(44.2)	2(3.8)		
Sex trade place					
Residence	7(26.9)	15(57.7)	4(15.4)	8.273	0.078
Hotel	58(40.0)	77(53.1)	10(6.9)		
Street	30(55.6)	21(38.9)	3(5.6)		
Monthly income in BDT					
< 8000	72(46.2)	71(45.5)	13(8.3)	4.516	0.105
≥ 8000	23(33.3)	42(60.9)	4(5.8)		

Only significantly associated factor, education level was considered as independent variable in multinomial logistic model to find the effect of this factor on level of FSWs’ knowledge about TB. This model demonstrated that secondary and higher educated FSWs were more likely to have fair knowledge than illiterate FSWs [OR = 0.075, 95%CI: 0.025–0.224; *p* < 0.01]. On the other hand, FSWs of secondary and higher education had more chance to have good knowledge about TB than the illiterate [OR = 0.064, 95%CI: 0.008–0.535; *p* < 0.01] and primary [OR = 0.136, 95%CI: 0.039–0.473; *p* < 0.01] educated FSWs (Table 9).

**Table 9:** Effect of education on the level of knowledge about TB among FSWs

Level of knowledge about TB		B	Wald	*P*-value	OR	95% CI for OR	
						LB	UB
Fair (3–4)	Intercept	0.936	12.589	0.001			
	Illiterate	−2.585	21.686	0.001	0.075	0.025	0.224
	Primary	−.785	5.727	0.017	0.456	0.240	0.868
	Secondary and Higher^R^	0	.	.	.	.	.
Good (5–6)	Intercept	−.511	1.957	0.162			
	Illiterate	−2.747	6.441	0.001	0.064	0.008	0.535
	Primary	−1.995	9.855	0.002	0.136	0.039	0.473
	Secondary and Higher^R^	0	.	.	.	.	.

NB: B: Coefficients, *OR* Odds ratio, *CI* Confidence interval, *LB* Lower bound, *UB* Upper bound and *R* Reference category

## Discussion

In the present study, general knowledge on TB among FSWs in Rajshahi city was investigated. May be this was the first time we attempted to survey knowledge on TB among FSWs in Rajshahi city, Bangladesh. This study revealed that more than 41% FSWs in Rajshahi city did not provide correct answer about type of TB disease, while only 9.8% FSWs heard about latent TB. A remarkable number of FSWs did not have clear idea about the main sign and symptom of TB while more than 76% FSWs did not know how TB spread. It was interested that still more than 21% FSWs believed that TB is incurable disease, also most of the FSWs (81.3%) did not have any idea about the most affected parts of the body by TB. Study on knowledge about TB among FSWs was not available; it was not possible comparing with other studies. However, researchers found that 71% primary school students in Malawi had knowledge about the type of TB disease [21]. It was found that the general knowledge on HIV/AIDS among FSWs in Rajshahi city, Bangladesh was very poor [2]. Education and family status (family income) were the most important predictors of the lack of general knowledge on any disease. In this study, it was noted that more than 63% FSWs did not complete their primary education and more than 69% FSWs lived with poor family (monthly income≤8000 Taka). This study also found that the FSWs who had secondary and higher level of education and monthly income of ≥8000 BDT had more knowledge regarding communicable disease than their counterpart. Female sex trade is illegal in Bangladesh but its demand is very high in the society and most of the time FSWs are very busy so they have no enough time to gather knowledge about the risks of communicable diseases like TB. WHO (2018) estimated that 23% (about 1.7 billion people) of the world’s population to have a latent TB infection. They are in the risk of developing active TB disease during their lifetime [22].

This latent TB has the chance of reactivation to active disease at any time when environment is favorable. In Chinese Immigrants in a Canadian a patient’ knowledge about latent TB was 19.8% [23] but present study found that FSWs knowledge about latent TB was only 9.8%. Limitation of access to TB screening facilities is very poor for FSWs compared to general population, because in day time most of the FSWs take rest and there is no chance of having TB screening at night time. Knowledge about transmission of TB in Nigerian population was 57.8% [24] but present study showed that FSWs’ knowledge on TB transmission was 23.6%. In Bangladesh, there is no special program of TB related issues exclusively for FSWs, though most of the respondents were known to have cough for 2 weeks or more, the main symptom of TB. Some study showed that most of the Indian students (86.0%) believed that TB was curable disease [25], this study demonstrated that 78.7% of the FSWs believed that TB was a curable disease. We found that the FSWs having secondary and higher level education and monthly income of ≥8000 BDT had more knowledge regarding TB is curable disease than their counterpart. The present study reported that FSWs’ education level, sex trade place, monthly family income, marital status were the important risk factors for the lack of general knowledge on TB among FSWs in Rajshahi city, Bangladesh, most of these factors had been found for the lack of knowledge on HIV/AIDS and FSWs in the same region [2]. This suggests that the FSWs with education and working in better social environment had better opportunity to learn about TB. Many governments, donor and non-government organizations (NGOs) were working for advocacy, communication and social mobilization (ACSM) in this city for general population but FSWs are not given special attention in this regard. To make the FSWs aware of TB, special measures like sessions, meetings, workshops, and video shows etc. regarding TB should be arranged in a regular manner especially with them.

### Study limitations

This study has some limitations. Firstly, the cross-sectional observational design did not permit us to establish any absolute chronological associations for identifying between knowledge on TB and various socio-demographic and health seeking behavior related features. Additional longitudinal research is desirable to fully identify this complex relationship and understand the underlying mechanisms. Secondly, this study used the only quantitative survey to FSWs knowledge on TB. For the development of culture-sensitive communication strategies, qualitative studies are also necessary. However, these approaches couldn’t be done due to time and resource constraints. We should consider this point in our future studies. Lastly, the idea of knowledge, which has several definitions, was hard to measure, especially using the questionnaire. However, this study measured the knowledge variables with several indicators which were used in some previous studies [21, 23–25].

## Conclusions

In the present study, we surveyed the general knowledge on TB among FSWs in Rajshahi city, Bangladesh, and data were collected from 225 randomly selected FSWs. Some questions were asked to FSWs for determining their general knowledge on TB, and it was observed that the knowledge of FSWs on TB was very poor. Moreover, we investigated the associated factors for lacking of TB knowledge among FSWs in Rajshahi city. It was observed that more than 42% FSWs had overall poor knowledge on TB while more than 50% had fair knowledge but only 7.6% FSWS had good knowledge on TB. It was found that comparatively more educated, living with rich family, currently unmarried, traded sex at residence, aged≥26 years FSWs were more knowledgeable than their counterparts. In conclusion, considering alarming situation revealed in this study regarding knowledge on TB of FSWs, the government and other concerned authorities should pay special attention to FSWs community as a vulnerable group. This study strongly recommends that advocacy, communication and social mobilization program is urgently needed for FSWs. It further suggests that access to TB screening and treatment facilities for the FSWs should be easy and immediately arranged.

## Data Availability

In this study database used for PhD study. So, available upon request sent to the corresponding author.

## Abbreviations

ACSM: Advocacy, communication and social mobilization
BDT: Bangladeshi Taka
FSWs: Female sex workers
IBSc: Institute of Biological Sciences
KMSS: Khulna Mukti Seba Sangstha
MDR: Multi drug resistance
NGO: Nongovernmental organization
RU: University of Rajshahi
TB: Tuberculosis
WHO: World health organization
